# Supplementary material for: Identification of Protosappanoside D from Caesalpinia decapetala and Evaluation of Its Pharmacokinetic, Metabolism and Pharmacological Activity
Source: Molecules. 2022 Sep 18;27(18):6090. doi: 10.3390/molecules27186090 (PMC9506044; doi:10.3390/molecules27186090)
Supplement: Supplementary file 1 [file molecules-27-06090-s001.zip › molecules-1902320-supplementary.pdf]

Supplementary Material S1

Metabolites were detected and identified in blood plasma samples. Full scans and product ion scans in positive ion mode were conducted for all samples. After comparison between blank and administered samples, four metabolites were identified.

Metabolite M1. The retention time of metabolite M1 was 4.55 min and its protonated molecule m/z of 303.0874 (3.1 ppm) was detected in positive ion mode. The elemental composition of M1 was $C_{16}H_{16}O_6^+$. This was 162 m/z lower than that of the parent ion. It was speculated that M1 caused desugarization of the PTD. In the product ion scan (Fig. S1 A), the fragmentation pattern of M1 showed one characteristic product ion at m/z 159.0442. These results suggested that metabolite M1 is a desugarization metabolite of PTD.

Metabolite M2. The retention time of metabolite M2 was 4.33 min and its protonated molecule m/z of 287.0906 (−3.4 ppm) was detected in positive ion mode. The elemental composition of M2 was $C_{16}H_{15}O_5^+$. This was 178 m/z lower than that of the parent ion. It was speculated that M2 underwent dealkylation and dehydration of PTD. In the product ion scan (Fig. S1 B), the fragmentation pattern of M2 showed two characteristic product ions at 257.0801 m/z and 239.0696 m/z. These results suggest that metabolite M2 is a dealkylated and dehydrated metabolite of PTD.

Metabolite M3. The retention time of Metabolite M3 was 4.60 min and its protonated molecule m/z of 285.0750 (−2.5 ppm) was detected in positive ion mode. The elemental composition of M3 was $C_{16}H_{13}O_5^+$. This was 180 m/z lower than that of the parent ion. It was speculated that M3 was formed by dealkylation, dehydration, and degradation of PTD. In the product ion scan (Fig. S1 C), the fragmentation pattern of M3 showed three characteristic product ions at 269.0801 m/z, 257.0801, and 239.0696 m/z. These results suggest that metabolite M3 is a dealkylation, dehydration, and degradation metabolite of PTD.

Metabolite M4. The retention time of metabolite M4 was 4.49 min and its protonated molecule m/z of 495.1485 (−2.6 ppm) was detected in the positive ion mode. The elemental composition of M4 was $C_{23}H_{27}O_{12}^+$. This was 30 m/z higher than that of the parent ion. It was speculated that M4 caused desaturation, oxidation, and methylation of PTD. In the product ion scan (Fig. S1 D), the fragmentation pattern of M4 showed three characteristic product ions at 301.0695 m/z, 283.0595, and 255.0641. These results suggest that metabolite M4 is a desaturation, oxidation, and methylation metabolite in PTD.

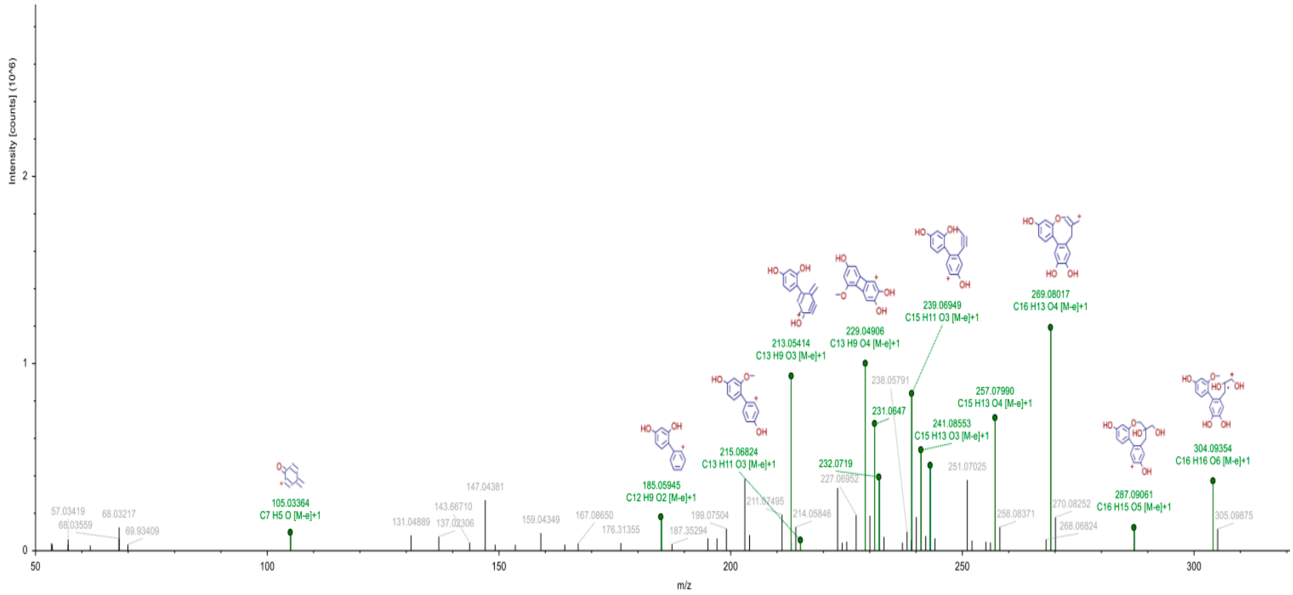

(A)

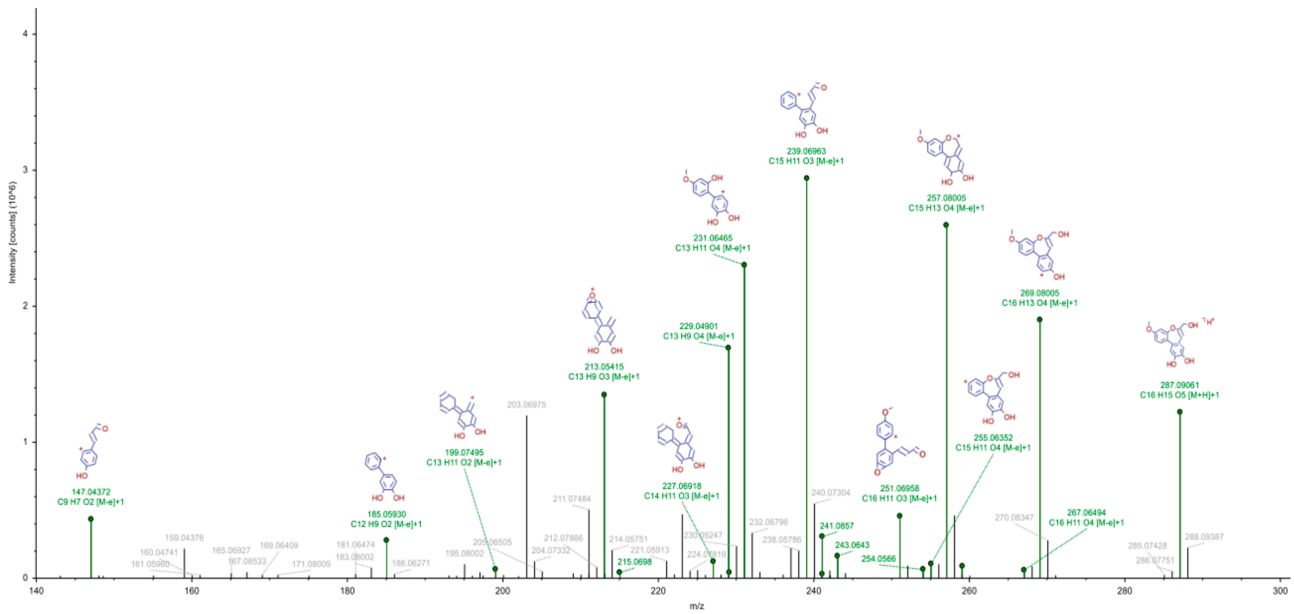
Intensity [counts] (10^6)
m/z
147.04372
C9 H7 O2 [M-e]+1
185.05930
C12 H9 O2 [M-e]+1
199.07495
C13 H11 O2 [M-e]+1
213.05415
C13 H9 O3 [M-e]+1
227.06918
C14 H11 O3 [M-e]+1
229.04901
C13 H9 O4 [M-e]+1
231.06465
C13 H11 O4 [M-e]+1
239.06963
C15 H11 O3 [M-e]+1
251.06958
C16 H11 O3 [M-e]+1
255.06352
C15 H11 O4 [M-e]+1
257.08005
C15 H13 O4 [M-e]+1
267.06494
C16 H11 O4 [M-e]+1
269.08005
C16 H13 O4 [M-e]+1
287.09061
C16 H15 O5 [M+H]+1

(B)

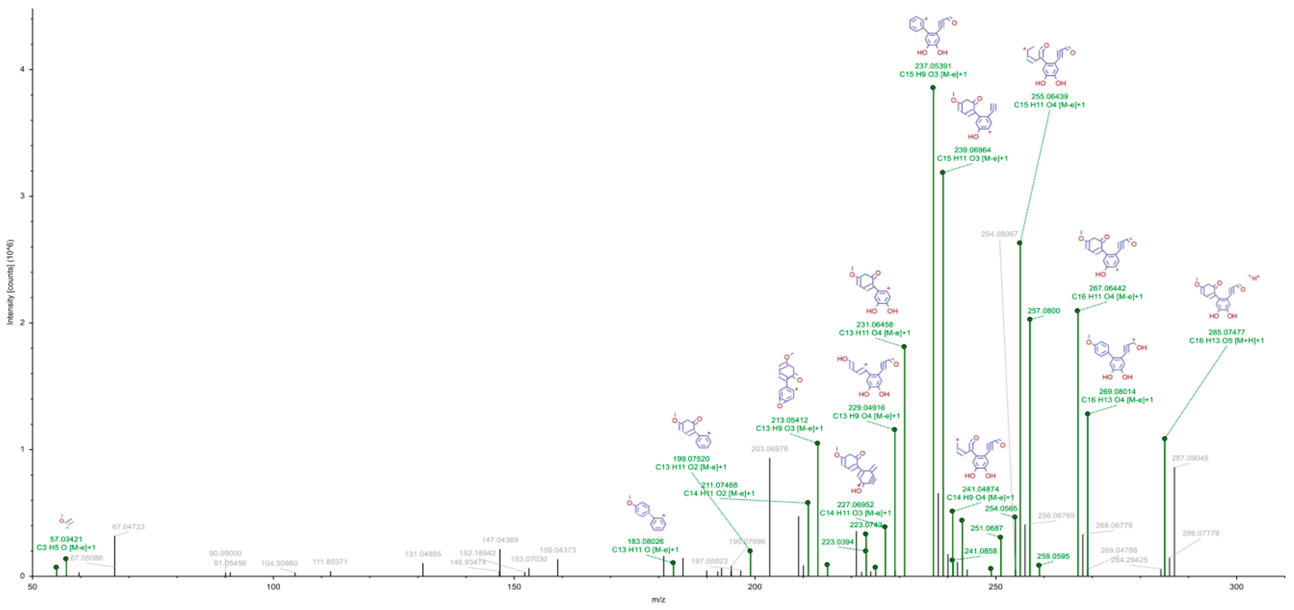
Intensity [counts] (10^6)
m/z
57.03421
C3 H5 O [M-e]+1
183.08026
C13 H11 O [M-e]+1
199.07520
C13 H11 O2 [M-e]+1
211.07468
C14 H11 O2 [M-e]+1
213.05412
C13 H9 O3 [M-e]+1
227.06952
C14 H11 O3 [M-e]+1
229.04916
C13 H9 O4 [M-e]+1
231.06458
C13 H11 O4 [M-e]+1
237.05391
C15 H9 O3 [M-e]+1
239.06964
C15 H11 O3 [M-e]+1
241.04874
C14 H9 O4 [M-e]+1
255.06439
C15 H11 O4 [M-e]+1
267.06442
C16 H11 O4 [M-e]+1
269.08014
C16 H13 O4 [M-e]+1
285.07477
C16 H13 O5 [M+H]+1

(C)

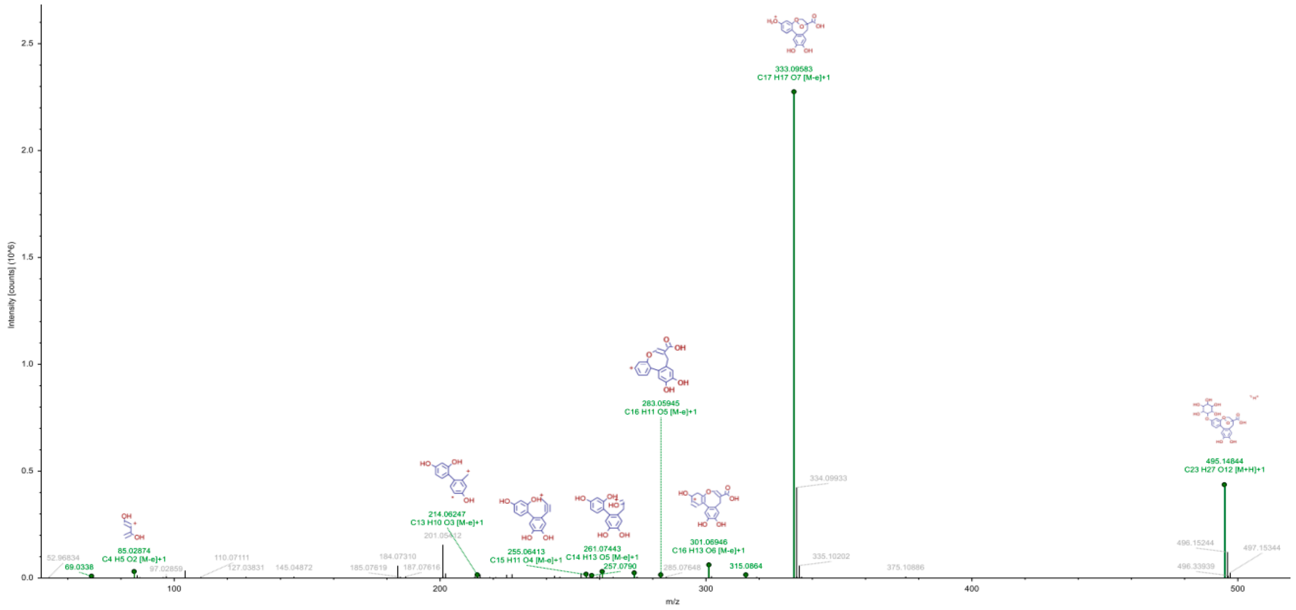

(D).

Figure S1. MS/MS spectrum of M1 (**A**), M2 (**B**), M3 (**C**) and M4 (**D**) in positive ion mode.

In this study, we described a strategy using UHPLC/Q Exactive Plus MS with Compound Discoverer 3.2 data analysis for the rapid analysis of PTD metabolites in rat plasma samples. PTD and the four metabolites were detected and tentatively identified simultaneously according to their retention time, accurate precursor ion masses, and fingerprint product ions. Metabolite conversion occurred via seven proposed pathways: dealkylation, dehydration, desaturation, oxidation, methylation, degradation, and desugation. UHPLC/Q Exactive Plus MS was used to analyse the biosamples, and data acquisition was performed in the positive ion mode.
